# Krüppel-like factor 17 inhibits urokinase plasminogen activator gene expression to suppress cell invasion through the Src/p38/MAPK signaling pathway in human lung adenocarcinoma

**DOI:** 10.18632/oncotarget.17020

**Published:** 2017-04-10

**Authors:** Xing-Dong Cai, Li Che, Jia-Xin Lin, Shuai Huang, Jiong Li, Xiao-Yan Liu, Xing-Fei Pan, Qin-Qin Wang, Li Chen, Ming-Juan Lin, Zhi-Hong Huang, Hong-Ming Ma, Yi Wu, Sheng-Ming Liu, Yan-Bin Zhou

**Affiliations:** ^1^ Department of Respiratory, The First Affiliated Hospital of Jinan University, Guangzhou 510630, China; ^2^ Department of Orthopedics, The Second Affiliated Hospital of Guangzhou Medical University, Guangzhou 510260, China; ^3^ Department of Anatomy, The Medical College of Jinan University, Guangzhou 510630, China; ^4^ Department of Infectious Disease, The Third Affiliated Hospital of Guangzhou Medical University, Guangzhou 510150, China; ^5^ Department of Pulmonary Medicine, The First Affiliated Hospital of Sun Yat-Sen University, Guangzhou 510080, China

**Keywords:** Krüppel-like-factor 17(KLF17), urokinase plasminogen activator (uPA), invasion, lung adenocarcinoma, signal pathway

## Abstract

Krüppel-like factor 17 (KLF17) has been reported to be involved in invasion and metastasis suppression in lung cancer, but the molecular mechanisms underlying the anti-invasion and anti-metastasis roles of KLF17 in lung cancer are not fully illustrated. Here, we showed that KLF17 inhibited the invasion of A549 and H322 cells; the anti-invasion effect of KLF17 was associated with the suppression of urokinase plasminogen activator (uPA/PLAU) expression. KLF17 can bind with the promoter of uPA and inhibit its expression. Enforced expression of uPA abrogated the anti-invasion effect of KLF17 in A549 and H322 cells. In addition, immunohistochemistry staining showed that the protein expression of KLF17 was negatively correlated with that of uPA in archived samples from patients with lymph node metastasis of lung adenocarcinoma (rho = −0.62, *P* = 0.01). The mutually exclusive expression of KLF17 with uPA could predict lymph node metastasis for lung adenocarcinoma (AUC = 0.758, *P* = 0.005). Enforced expression of KLF17 inhibited the expression of phosphorylated Src and phosphorylated p38/MAPK in A549 and H322 cells. The invasiveness of the cells were suppressed by treating with sb203580 (p38/MAPK inhibitor) or HY-13805 (PP2, Src inhibitor). furthermore, p38/MAPK inhibition could block the KLF17-induced reduction of p-p38/MAPK and uPA, and Src inhibition enhanced the KLF17-induced suppression of p-Src and uPA in A549 and H322 cells. In conclusion, our study indicated that KLF17 suppressed the uPA-mediated invasion of lung adenocarcinoma. The Src and p38/MAPK signaling pathways were suggested as mediators of KLF17-induced uPA inhibition, thus providing evidence that KLF17 might be a potential anti-invasion candidate for lung adenocarcinoma.

## INTRODUCTION

Worldwide, lung cancer is the leading and the second-leading cause of cancer-related death among men and women, respectively [1]. In 2014, approximately 220,000 cases of lung cancer were diagnosed, and 160,000 patients died due to lung cancer in the United States [2]. Approximately 40% of patients newly diagnosed with lung cancer were diagnosed at a stage of extensive local invasion and metastasis, and their 5-year survival rate is only was 1% [3, 4]. Lung adenocarcinoma (LCA) is the most common among the three major histologic types of non-small cell lung cancer (NSCLC), and LCA has a high metastasis potential compared to the other histologic types of NSCLC [5, 6]. Cancer cell invasion and metastasis are the major causes of death in patients with lung adenocarcinoma, but the mechanisms are largely unknown. Thus, it is of great significance to clarify the mechanisms of invasion and metastasis for the early diagnosis and treatment of lung cancer.

Urokinase plasminogen activator (uPA/PLAU) was originally found in urine, thus it is also known as the urinary-type plasminogen activator [7]. Newly synthesized single-stranded uPA is rapidly transformed into double-stranded uPA by plasmin or kallikrein. Both single and double-stranded uPA can bind to the urokinase plasminogen activator receptor (uPAR) [8], which significantly enhances the expression of uPAR mRNA and protein [9]. This combination greatly enhances the ability of uPA activating plasminogen and results in extracellular matrix protein degradation. The activities of fibroblast growth factor and vascular endothelial cell growth factor were increased due to uPA binding with uPAR; transforming growth factor beta (TGF beta), which regulates cell growth and differentiation, is also activated [10]. In addition to the activation of the fibrinolytic enzyme, the binding of uPA with uPAR can also activate collagenase, and thus, play an important role in tumor growth, cell migration and metastasis. The expression of uPA and its receptor uPAR were increased significantly in breast cancer, ovarian cancer, gastric cancer, esophageal cancer, lung cancer, liver cancer, colon cancer, prostate cancer and bile duct cancer [11–17]. Overexpression of uPA is a major predictor of poor prognosis in these patients, and the tumor cells with uPA overexpression frequently showed malignant invasion and a metastasis phenotype. Downregulation of uPA and its receptors can inhibit tumor cell growth, invasion and survival [18]. The uPA/uPAR system is involved in tumorigenesis and tumor progression, including tumor cell proliferation, migration, adhesion, angiogenesis and invasion [19]. The signaling pathways of p38/MAPK [20], NF kappa B [21], and PI3K/AKT [22] participate in the regulation of uPA promoter activity, which upregulates the expression of uPA. The regulatory mechanism of uPA expression has not been clarified so far, particularly in lung adenocarcinoma.

As seen in the overexpression of uPA in various human cancers, Krüppel-Like Factor 17 (KLF17) is also downregulated in human cancers, including lung cancer [23], liver cancer [24], gastric cancer [25], papillary thyroid carcinoma [26], esophageal carcinoma [27], and colorectal carcinoma [28], and reduced expression of KLF17 is associated with poor prognosis in these patients. Human KLF17, a new member of the Sp/KLF family of transcription factors, has been reported to bind to the G/C-rich sites of target genes via its zinc fingers [29]. KLF17 inhibits epithelial-mesenchymal transition (EMT) and invasion of breast cancer cells, and KLF17 protein directly inhibits the transcription of Id1, the key regulator of the metastasis of breast cancer [30]. In addition, KLF17 suppresses the invasion of cancer cells through its interactions with CD44, PAI-1, Cyclin-D1 [31]. In the present study, we found that KLF17 suppressed lung adenocarcinoma cell invasion partially by negatively regulating uPA. A dual-luciferase reporter assay also showed that KLF17 could bind to the promoter of uPA. IHC analysis also showed that KLF17 was negatively correlated with uPA expression in patients with lymph node metastasis of lung adenocarcinoma. Furthermore, KLF17 inhibited the expression of Phospho- p38/MAPK and Phospho-Src, the inhibition of p38/MAPK blocked the KLF17-induced suppression of uPA, and the inhibition of Src enhanced the KLF17-induced suppression of uPA, therefore suggests that KLF17 suppress the expression of uPA to inhibit lung adenocarcinoma invasion via the p38/MAPK and Src pathways.

## RESULTS

### KLF17 protein suppressed the invasiveness of A549 and H322 cells

**Figure 1:**
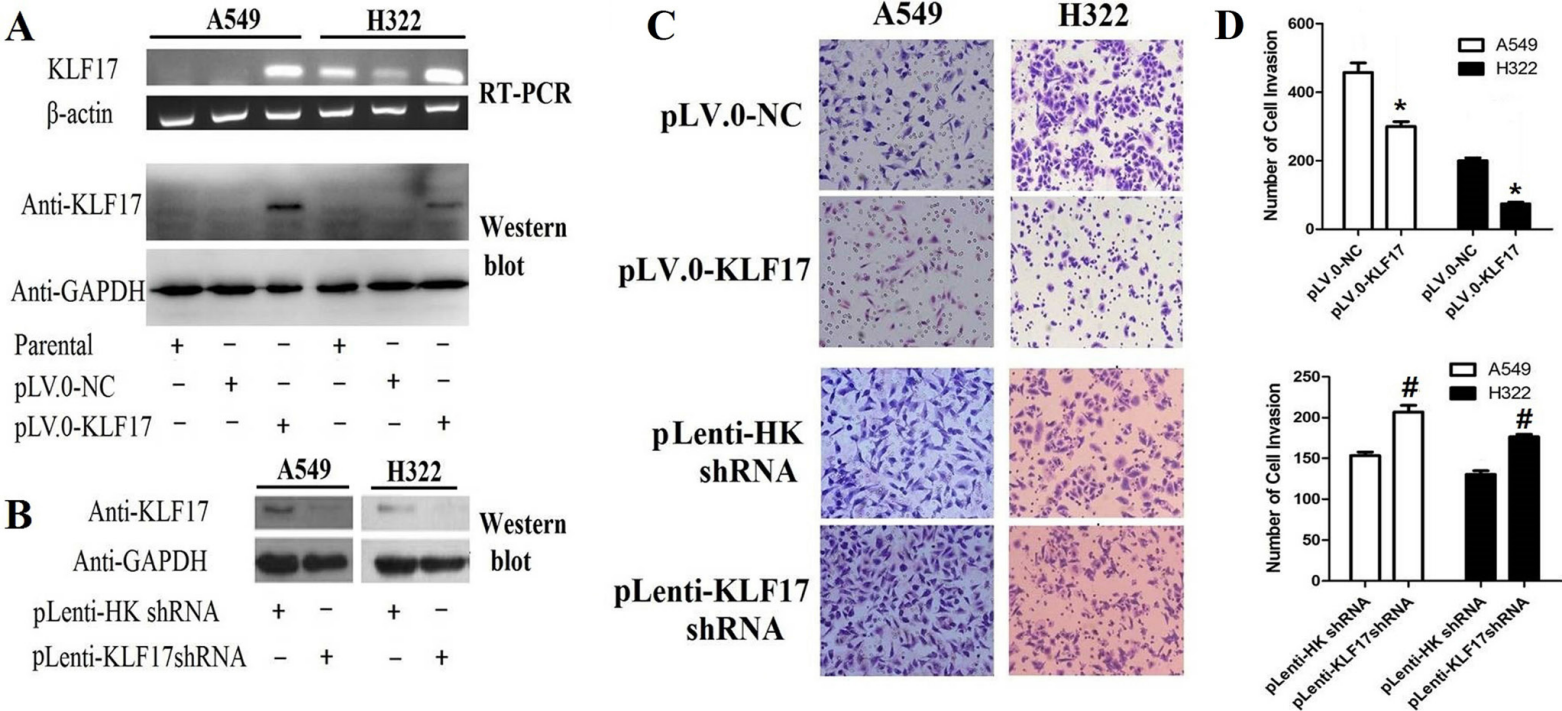
Overexpression of KLF17 inA549 and H322 cells inhibited the invasiveness of lung adenocarcinoma cells (**A**, **B**) Using RT-PCR and Western blot, we found that KLF17 expression was increased in A549 and H322 cells infected with lentiviral particles containing pLV.0-KLF17, while the KLF17 protein was decreased in A549 and H322 cells infected with pLenti-KLF17 shRNA compared to the control(empty vector) groups; (**C**) Representative photomicrographs of invaded cells(×200) showed that the overexpression of KLF17 inA549 and H322 cells inhibited the invasiveness of the cells compared to that of the control groups. Downregulation of KLF17 in A549 and H322 enhanced the invasiveness of the cells compared to the control groups; (**D**) The graphs show the number of invaded cells/field in five Transwells(under×200 original magnification). Quantitative data represent means and SD of three independent experiments. (^*#^*P* < 0.05).

KLF17 can suppress the invasiveness of breast cancer cells [32], hepatocellular carcinoma [24], and esophageal carcinoma [27] and has been correlated with prognosis in these patients. In the present study, we examined the effect of KLF17 protein on the invasiveness of A549 and H322 cells, as shown in Figure 1. The A549 and H322 cells were infected with lentiviral particles containing KLF17 cDNA (pLV.0-KLF17) and control Lentiviral particles (pLV.0-NC). RT-PCR and Western blot analysis showed that KLF17 was increased at both the mRNA and protein level compared to the control groups or parental groups, therefore indicating that KLF17 was stably overexpressed in A549 and H322 cells. Then, we stably decreased KLF17 expression using pLenti-KLF17 shRNA in a similar manner (Figure 1A). Next, we examined the effect of KLF17 on the invasiveness of A549 and H322 cells using a Transwell assay. The number of invaded cells were decreased dramatically in the A549 and H322 cells infected with pLV.0-KLF17 compared to the cells infected with pLV.0-NC (*P* < 0.05). The invasiveness of A549 and H322 cells infected with pLenti-KLF17shRNA was increased significantly compared to the cells infected with pLenti-HK shRNA (*P* < 0.05) (Figure 1B, 1C), indicating that KLF17 suppressed the invasion of lung adenocarcinoma cells.

### KLF17 protein suppressed the expression of uPA in A549 and H322 cells by binding with the promoter of uPA

**Figure 2:**
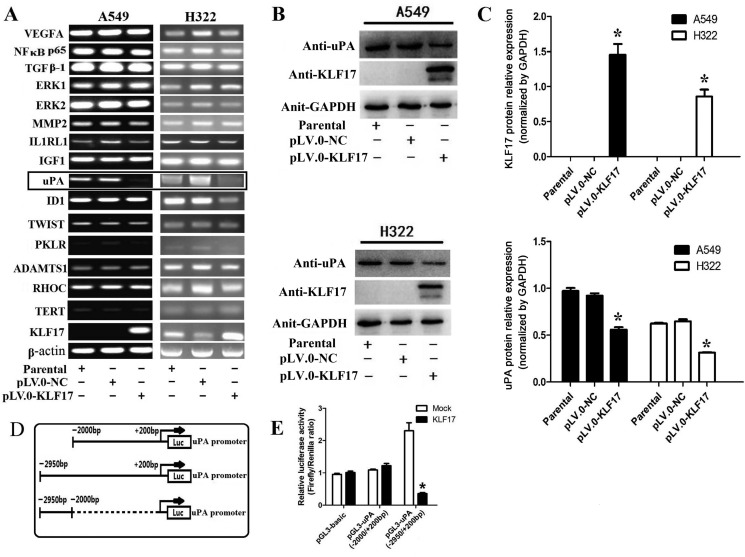
Enforced expression of KLF17 inA549 and H322cells inhibited the expression of uPA by binding with its promoter (**A**) The expression of MMP2, ERK1/2, TGFβ1, NFκBp65, VEGFA, TWIST, Id1, IGF-1, IL1RL1, TERT, RHOC, ADAMTS1, and PKLR was not changed after the upregulation of KLF17 in A549 and H322 cells, but the expression of uPA was reduced significantly when KLF17 was upregulated in A549 and H322 cells (displayed in box); (**B**) Western blot analysis showed that uPA protein expression was reduced when KLF17 protein was upregulatedin A549 and H322 cells. GAPDH was used as a loading control; (**C**) The graphs showed that the KLF17 protein was expressed at higher levels in A549 and H322 cells infected with lentiviral particles containing pLV.0-KLF17 than the parental cells or the cells infected with lentiviral particles containing pLV.0-NC (empty) (*P < 0.05). (**D**) A schematic representation of potential binding sequences in the uPA promoter. (**E**) The luciferase vectors containing different sequences were co-transfected with pcDNA3.1(+)-KLF17 or mock vector in 293FT cells for 48 h and detected by a luciferase assay. Quantitative data represent means and SD of three independent experiments (*P < 0.05).

To further investigate the role of KLF17 in the invasiveness of lung adenocarcinoma, we analyzed the expression of invasion-related tumor-cell genes, including MMP2, ERK1/2, TGFβ1, NFκBp65, VEGFA, TWIST, Id1, IGF-1, IL1RL1, TERT, RHOC, ADAMTS1, PKLR and uPA [33], between the KLF17-overexpression group and the control or parental groups. Of interest, the results showed that the uPA mRNA expression was decreased significantly in A549 and H322 cells infected with lentiviral particles containing pLV.0-KLF17 (KLF17 overexpression) compared to cells infected with lentiviral particles containing pLV.0-NC and the parental cells. Id1 has been reported as a negatively regulated gene by KLF17 in breast cancer. However, in our study, Id1 mRNA expression was decreased only in H322 cells that overexpressed KLF17. Whereas the mRNA levels of most of the other genes were not extensively changed (Figure 2A). Western blot analysis also showed that the KLF17 protein was expressed at higher levels in A549 and H322 cells infected with lentiviral particles containing pLV.0-KLF17 than the parental cells or the cells infected with lentiviral particles containing pLV.0-NC (empty control). As expected, the ectopic expression of KLF17 led to the downregulation of uPA protein in A549 and H322 cells (Figure 2B, 2C). For further validation of whether uPA expression was suppressed by KLF17 at a transcriptional level, luciferase vectors containing different sequences were co-transfected with pcDNA3.1(+)-KLF17 or mock vectors in 293FT cells for 48 h, and then, they were detected by a dual-luciferase reporter assay, and the results showed that the promoter sequence (−2950 to −2000) was bound by the KLF17 protein, but not in the −2000 to +200 region. These data demonstrated that KLF17 protein could combine with the uPA promoter sequence at (−2950 to −2000) and inhibited the expression of uPA (Figure 2D and 2E).

### KLF17 protein inhibited the invasion of A549 and H322 cells through the suppression of uPA

**Figure 3:**
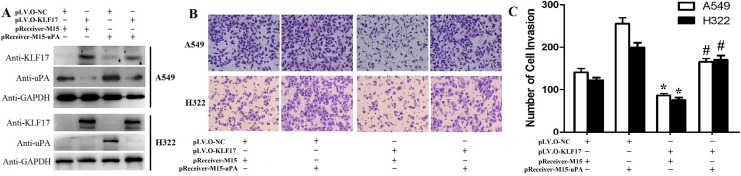
Enforced expression of uPA restored the KLF17-mediated anti-invasion effects in A549 and H322 cells (**A**) Western blot analysis shows that enforced expression of uPA in A549 and H322 cells where KLF17 is also stably upregulated significantly increased uPA protein expression. GAPDH was used as a loading control. (**B**) Representative areas of invaded cells by the different A549 and H322 cells on the Transwell assay. (**C**) The graphs show the number of invaded A549 or H322 cells/field. Quantitative data represent means and SD of three independent experiments. *P value < 0.01 compared with uPA up-regulation group; ^#^*P* value < 0.05 compared with KLF17 up-regulation group.

The above experiments showed that KLF17 protein could inhibit the invasion of A549 and H322 cells and that KLF17 protein suppressed the expression of the cancer invasion-related gene uPA. Next, we investigated the KLF17-mediated anti-invasion activities and whether or not it was associated with the inhibiton of uPA. The stably KLF17 protein-expressing cells were transfected with a uPA overexpression vector, and their *in vitro* invasion ability was evaluated by a Transwell assay. Western blot analysis confirmed that ectopically enhanced expression of uPA in KLF17-overexpression cells significantly increased uPA expression as compared with the cells transfected with an empty vector (Figure 3A). The Matrigel-based Transwell assay demonstrated that ectopically enforced expression of uPA significantly increased the number of invaded cells compared to the control groups (empty vector) in A549 and H322 cells of the KLF17-negative groups. Meanwhile, in the KLF17-overexpression groups, the invaded cells were remarkably decreased compared to the control groups, and uPA overexpression significantly increased the number of invaded cells, therefore suggesting that the enforced expression of uPA restored the KLF17-mediated anti-invasion activity in A549 and H322 cells (Figure 3B and 3C). These data illustrated that the KLF17-induced inhibition of uPA expression is vital for the KLF17-mediated suppression of lung adenocarcinoma cell invasion.

### KLF17 expression was negatively correlated with the expression of uPA in patients with lung adenocarcinoma

**Figure 4:**
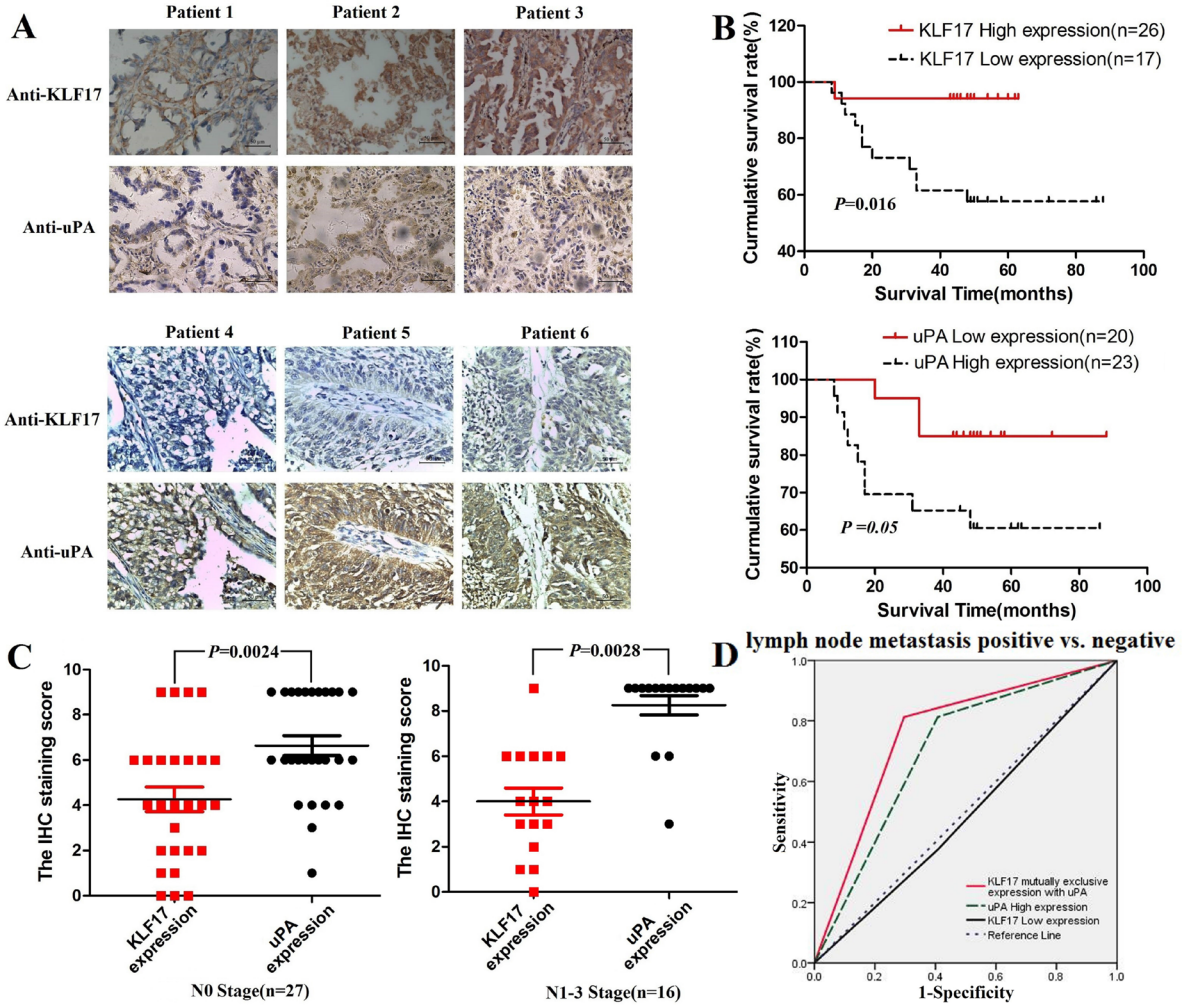
The expression of KLF17 in lung adenocarcinoma tissues was negatively correlated with the expression of uPA and was correlated with the overall survival time of patients (**A**) Representative images from 43 cases of lung adenocarcinoma with KLF17 or uPA immunostaining (400×), scale bar: 50 μm; (**B**) The overall survival time of patients with lung adenocarcinoma was significantly different between the high-KLF17/low-uPA and low-KLF17/high-uPAexpression groups (KLF17, *P* < 0.05; uPA, *P* = 0.05); (**C**) The difference in the KLF17 and uPA immunostaining score between the lymph node metastasis positive and negative groups (*p* < 0.05). (**D**) ROC curve showing that KLF17 is mutually and exclusively expressed with uPA (AUC = 0.758, *P* = 0.005), uPA high expression alone (AUC = 0.703, *P* = 0.028), and KLF17 low expression alone (AUC = 0.484, *P* = 0.86) for the patients that are lymph node metastasis positive *vs*. patients that are lymph node metastasis negative.

*In vitro* analysis indicated that KLF17 inhibited the invasion of lung adenocarcinoma cells at least partially through the suppression of uPA. We then evaluated the correlation between the expression of KLF17 and uPA in tumor tissues from 43 cases of lung adenocarcinoma by immunohistochemical staining and analyzed the expression statuses of KLF17 and uPA in N stage. First, we calculated the optimal cut-off value for the immunostaining score by analyzing the correlation between KLF17 or uPA expression and the survival time of the patients with lung adenocarcinoma. The results showed that the optimal cut-off value for the high expression of KLF17 or uPA was ≥ 6 and ≥ 9, respectively. Log-rank analysis illustrated that the overall survival time was significantly different between the High-KLF17/Low-uPA and Low-KLF17/High-uPA expression groups (KLF17, *P* < 0.05; uPA, *P* = 0.05). Reduced expression of KLF17 and overexpression of uPA were associated with a short survival time in patients with lung adenocarcinoma (Figure 4B). Wilcoxon rank-sum test analyzed the differences between immunostaining scores of KLF17 and uPA expression in the lymph node metastasis-positive or -negative groups. The differences were all observed and were inclined to be significant in the lymph node metastasis-positive group (Figure 4C). The results illustrated that the patients with reduced expression of KLF17 and increased expression of uPA in lung cancer were more likely to have lymph node metastasis. Receiver operator characteristic curve (AUC) analysis additionally showed that the mutually exclusive expression of KLF17 with uPA could predict lymph node metastasis in lung adenocarcinoma, the sensitivity and specificity for diagnose to lymph node metastasis was 81.2% and 71.4% respectively, and the AUC = 0.758 (*P* = 0.005), making it a better predictive factor than uPA overexpression alone (sensitivity = 81.2%, specificity = 59.3%, AUC = 0.703, *P* = 0.028) or low KLF17 expression alone (sensitivity=37.5%, specificity = 59.6%, AUC = 0.484, *P* = 0.86) (Figure 4D). Furthermore, Spearman analysis uncovered that the expression of KLF17 was negatively correlated with that of uPA only in the lymph node metastasis positive groups (rho = −0.62, *P* = 0.01). These results demonstrated that KLF17 suppresses the expression of uPA in the invasion and metastasis of the lung adenocarcinoma (Tables 1 and 2).

**Table 1:** KLF17 and uPA expression in tumor tissues from 43 cases oflung adenocarcinoma

uPA expression (*n*)	KLF17expression(*n*)		rho value	*P* value
	High(*n*)	Low(*n*)		
High(*n*)	10	14	0.049	0.75
Low (*n*)	7	12		

**Table 2:** KLF17 and uPA expression in tumor tissues from 16 cases of lung adenocarcinoma and lymph node metastasis

uPA expression (*n*)	KLF17expression(*n*)		rho value	*P* value
	High (*n*)	Low (*n*)		
High (*n*)	3	10	−0.62	0.01
Low (*n*)	3	0		

### KLF17 inhibited the expression of uPA by repressing the p38 MAPK/Src signaling pathway

**Figure 5:**
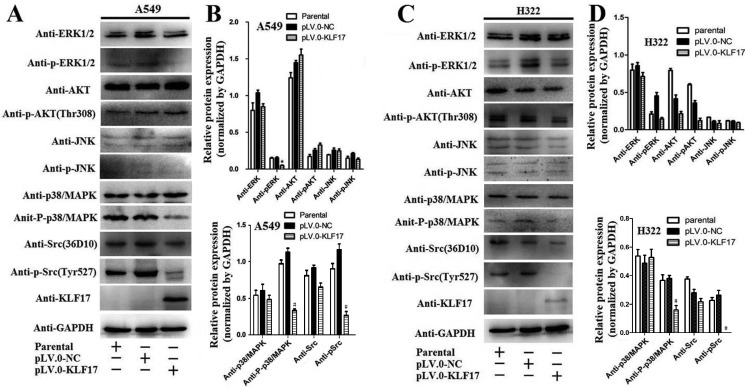
KLF17 inhibited the expression of phosphorylated-p38/MAPK and phosphorylated-Src (**A, C**) Western blot analysis showed that the overexpression of KLF17 in A549 and H322 cells significantly decreased p-Src and p-p38/MAPK protein expression when compared to the cells infected with pLV.0-NC or the parental cells. But p-ERK was decreased in A549 cells but not in H322 cells in the KLF17-overexpression groups. (**B, D**) The histograms show that the p-Src and p-p38/MAPK protein levels were downregulated in A549 and H322 cells infected with lentiviral particles containing pLV.0-KLF17 compared to the parental cells or the cells infected with lentiviral particles containing pLV.0-NC (empty). Meanwhile, the p-AKT and p-JNK were not altered in the three groups. The experiments were repeated in triplicate, and the data are represented as the mean ± s.d. (*P < 0.05).

There is increasing evidence indicating that uPA was activated by the p38/MAPK [34–36] and Src signaling pathways [37, 38], and inhibiton of the p38/MAPK and Src also supress the invasion of cancer cells [39–41]. To elucidate the mechanisms underlying the KLF17 inhibition of uPA in invasion of lung adenocarcinoma, we analyzed whether or not KLF17 participated in the Src, p38/MAPK, JNK, AKT or ERK pathways, and the results demonstrated that the phosphorylation of Src and p38/MAPK was significantly decreased in the KLF17-overexpression groups (pLV.O-KLF17) compared to the control groups (pLV.O-NC) or parental groups in A549 and H322 cells (Figure 5A–5D, *p* < 0.05). Meanwhile, the phosphorylation of ERK proteins was lower in the KLF17-overexpression groups than the control groups or the parental groups in A549 cells, but not in H322 cells, which suggested that the involvement of KLF17 in the ERK pathway is cell-content specific. These results illustrated that KLF17 could inhibit the activities of the Src and p38/MAPK signaling pathways.

**Figure 6:**
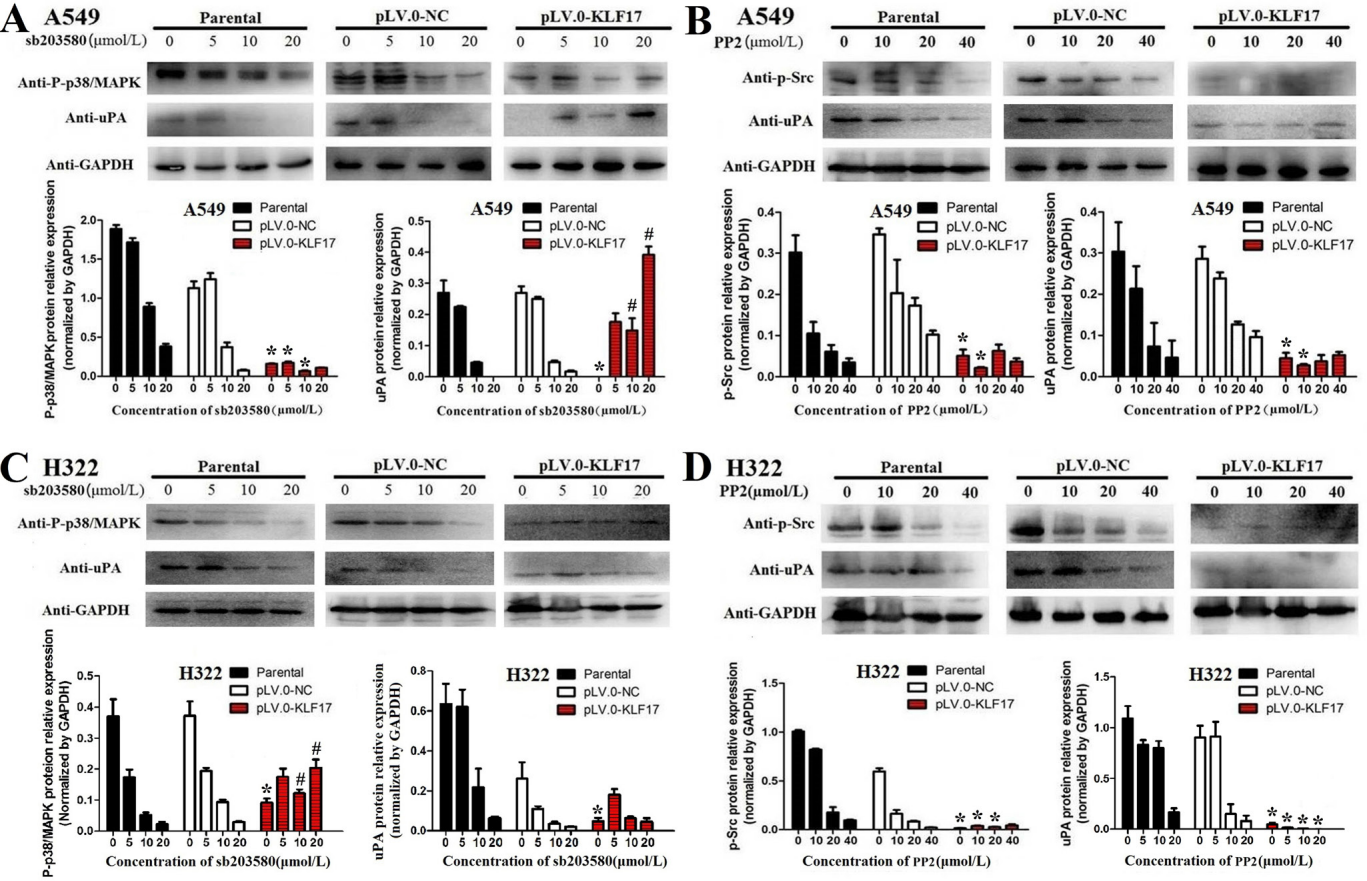
KLF17 negatively regulated the expression of uPA via the suppression of the Src and P38/MAPK signaling pathways (**A, B**) Western blot and histograms show the p-p38/MAPK or uPA protein expression in the parental, NC control and KLF17-overexpression groups when the A549 and H322 cells were treated with different concentrations of SB 203580 (p38/MAPK inhibitor). (**C, D**) Western blot and histogram show the p-Src or uPA protein expression in the parental, NC control and KLF17-overexpression groups when the A549 and H322 cells were treated with different concentrations of HY-13805 (Src inhibitor). The experiments were repeated at least in duplicate, and the data are represented at the mean ± s.d. ^*#^*P* value < 0.01 compared with NC group and parental group.

We then used SB 203580 (p38/MAPK inhibitor) or HY-13805 (PP2, Src inhibitor) to treat the A549 and H322 cells for 30 min and 2 h, respectively, and the cells were subsequently subjected to the invasion and immunoblotting assay. The invasion asssy showed that inhibition of p38/MAPK and Src suppressed the invasion of A549 and H322 cells in a dose-dependent manner (Supplementary Figure 1). The western blot analysis showed that p38/MAPK inhibition led to a decrease in the expression of p-p38/MAPK or uPA in a dose-dependent manner in the parental or control groups, but p38/MAPK inhibition could block the KLF17-induced reduction of p-p38/MAPK and uPA in the KLF17-overexpression groups. The inhibition of Src also led to reduction of p-Src and uPA in a dose-dependent manner in the parental or control groups, and Src inhibition enhanced the KLF17-induced suppression of p-Src and uPA in the KLF17-overexpression groups of the A549 and H322 cells. (Figure 6A–6D). However, only inhibition of p38/MAPK and Src could not suppress the uPA at transcriptional level (Supplementary Figure 2). These results illustrated that KLF17 inhibits the activity of p38/MAPK or Src and then induces uPA suppression, thereby inhibiting the invasion of lung cancer cells.

## DISCUSSION

The present study demonstrated that KLF17 suppressed the invasion of lung adenocarcinoma cells, and the anti-invasion effects of KLF17 on cancer cells were caused partially by inhibiting the expression of uPA, which has been reported to be involved in crucial cellular functions of invasion and metastasis of breast cancer, ovarian cancer, gastric cancer, esophageal cancer, lung cancer, liver cancer, colon cancer, prostate cancer and bile duct cancer [11–17]. Ectopic upregulation of uPA impaired the anti-invasion activity of KLF17 in lung adenocarcinoma cells. Moreover, a dual-luciferase reporter assay showed that the KLF17 protein can bind to the promoter of uPA. Immunohistochemical staining also showed that reduced expression of KLF17 and overexpression of uPA were both correlated with poor prognosis in patients with lung adenocarcinoma. KLF17 protein expression was negatively correlated with uPA protein expression in tumor tissues from 16 cases of lung adenocarcinoma with lymph node metastasis (rho = −0.62, *P* = 0.01). ROC curve analysis showed that the mutual and exclusive KLF17 expression with uPA is predictive for lymph node metastasis in patients with lung adenocarcinoma (AUC = 0.758, *P* = 0.005). These results indicated that KLF17 inhibited lung adenocarcinoma cell invasion at least partially by the suppression of uPA gene expression.

KLF17 is a new member of the Krüppel-Like Factors family and has been reported to bind to G/C rich gene sequences by its zinc finger to activate the transcription of CACCC-box elements. Recently, accumulating evidence has shown that KLF17 is downregulation in lung cancer [23], liver cancer [24], gastric cancer [25], papillary thyroid carcinoma [26], esophageal carcinoma [27], and colorectal carcinoma [28]; KLF17 downregulation increased the invasiveness of these cancers and was correlated with poor prognosis in these patients. The anti-invasion role of KLF17 has been reported to regulate Id1 expression in breast cancer [30]. However, in our study, KLF17 inhibited Id1 expression only in H322 cells, not in A549 cells, possibly due to KLF17's tissue-specific expression and the diversity of post-transcriptional regulation [42]. Interestingly, KLF17 inhibited uPA expression in A549 and H322 cells at the mRNA and protein levels. In addition, we used a dual-luciferase reporter assay to confirm that KLF17 could bind to the promoter of uPA and suppress its expression. These data suggest that KLF17 inhibits uPA expression in lung adenocarcinoma. Furthermore, the Matrigel-based Transwell assay illustrated that enforced expression of uPA in A549 and H322 cells in which KLF17 was stably upregulated significantly reversed the anti-invasion effects of KLF17 in these cells. These results confirmed that the anti-invasion effect of KLF17 in A549 and H322 cells was associated with the inhibition of uPA. The results from the immunohistochemical staining analysis showed that the expression of KLF17 was not negatively correlated with uPA expression in lung adenocarcinoma tissues unless the patients had lymph node metastasis. However, KLF17 promotes the invasion of endometrioid endometrial cancer (EEC) and was observed to be elevated with TWIST1 in EEC tissues [43], which implies that it may have roles in other complex regulatory mechanisms including suppressive pathways.

Ali, A et al. [44] investigated the signaling pathways that interact with KLF17 in the suppression of tumor progression, and the results showed that KLF17 plays an integral role in potentiating TGF-beta/Smad signaling via the Smad3-dependent pathway, thus providing a new model of signal pathway regulation by KLF17 in the progression of invasion or metastasis of tumor cells. In our study, we found that enforced KLF17 expression in A549 and H322 cells significantly inhibited phospho-p38/MAPK and phospho-Src expression compared to the control groups and parental groups. We also investigated if KLF17 was involved in the JNK, AKT and ERK pathways in A549 and H322 cells, and the results showed that the phosphorylation of JNK and AKT was not altered in the KLF17-overexpression, control or parental groups. However, phospho-ERK was decreased in A549 cells but not in H322 cells in the KLF17-overexpression groups, which suggests that KLF17 could participate in the supression of the P38/MAPK and Src signaling pathways and could inhibit the ERK signal pathway depending on the cell context. Furthermore, we used SB 203580 (p38/MAPK inhibitor) or HY-13805 (Src inhibitor) to treat the A549 and H322 cells, and the transwell assay showed that p38/MAPK and Src inhibition significantly suppressed the invasiveness of A549 and H322 cells, consisting with the results from Gautschi, O et al [39] and Kato, S et al. [41]. these result illustrated that KLF17 supressed invasiveness of A549 and H322 cells by inhibition the P38/ MAPK and Src signaling pathways.

Increasing numbers of studies have demonstrated that uPA is regulated by the p38/MAPK signaling pathway in the invasion of cervical [45], breast [46] and colon cancer cells [36]. Malinowsky, K et al. [47]evaluated the roles of uPA and PAI-1 in thePI3K/AKT and MAPK pathways in the primary tumors and metastases of breast cancer, and the results illustrate that there is good correlation between uPA and p38/MAPK in tumors and metastases, and these results demonstrate that uPA is involved in cancer invasion and metastasis partially through the activation of the p38/MAPK pathway. However, uPA also promotes p38 phosphorylation and enhances the migratory and invasive capabilities of endometrial tumor cells [48]; therefore, the regulatory mechanisms between uPA and p38 are complicated and need further investigation. In the present study, we found that enforced expression of KLF17 in A549 and H322 cells significantly suppressed the expression of p-p38/MAPK in these cells. We also used SB 203580 (p38/MAPK inhibitor) to treat the A549 and H322 cells, and the results showed that p38/MAPK inhibition leads to a decrease in p-p38/MAPK or uPA expression in a dose-dependent manner in the parental or control groups. Interestingly, p38/MAPK inhibition could block the KLF17-induced reduction of p-p38/MAPK and uPA in the KLF17-overexpression group, which suggests that p-p38/MAPK and uPA are downstream target molecules of KLF17 and that the p38/MAPK pathway is required for KLF17 to suppress uPA in lung adenocarcinoma.

Recent studies suggest that the Src pathway is aberrantly activated in various types of cancers and is involved in cell division, motility, adhesion, and angiogenesis in the cancers [49]. Src is usually activated through structural alterations mediated by upstream kinases or phosphatases. uPA could be a downstream target protein of Src [50]. In our study, we used HY-13805(a Src inhibitor) to treat A549 and H322 cellsand then analyzed the expression of p-Src and uPA by Western blot. The results indicated that Src inhibition results in the reduction of p-Src and uPA significantly in a dose-dependent manner in the parental and control groups, showing that KLF17 inhibits the expression of uPA via the Src signaling pathway. In addition, Src inhibition enhanced the KLF17-induced suppression of p-Src and uPA in the KLF17-overexpression groups of A549 and H322 cells. These findings are consistent with the previous studies and confirm that KLF17 suppresses the p-Src/uPA cascade, and KLF17 inhibition of uPA is partially dependent on the Src signaling pathway.

In conclusion, our study demonstrated that KLF17 inhibited the invasion of lung adenocarcinoma cells, and the effects were correlated with the suppression of uPA expression. In addition, increased expression of uPA and reduced expression of KLF17 were prognostic indicators for survival time of patients with lung adenocarcinoma, and KLF17 was negatively correlated with uPA expression in the tumor tissues from patients with lymph node metastasis of lung adenocarcinoma. KLF17 inhibited uPA through the SRC/P38/MAPK pathway. Our study provides a new model for the negative regulation of uPA by KLF17 in mediating the invasion of lung adenocarcinoma via the SRC/P38/MAPK pathway. In conclusion, KLF17 might be a potential anti-invasion candidate in the treatment of lung adenocarcinoma.

## MATERIALS AND METHODS

### Cell culture,transient infection and lentivirus infection

Lung cancer cells A549 and H322 were cultured as previously described in our study [23]. Lentivirus vector pLV.0-KLF17 containing the human KLF17 coding sequence(NM_173484;1170 bp) or the control empty vector (pLV.0-NC) were purchased from GeneCopoeia (MD). The KLF17 shRNA Lentivirus vector (GV248, target sequences:5′-CGACAGTACCTTCTGACGAA AC-3′, pLenti-KLF17shRNA) and their control vector (control sequence:5′-TTCTCCGAACGTGTCACGT-3′, pLenti-HK shRNA) were obtained from GeneChen Inc. (Shanghai, China). The lentivirus vector contains EGFP and puromycin as selection markers for mammalian cell lines. The vectors were cotransfected into 293T cells with lentiviral packaging vectors according to the manufacturer's instructions. Subsequently, the infectious lentiviral particles were collected from the supernatant of the 293T cells at 48 to 72 h after cotransfection. A549 and H322 cells were infected with these lentiviral particles and selected with puromycin (Invitrogen Corporation, Carlsbad, CA) for 2 weeks. The regulatory efficacy of KLF17 was confirmed by RT-PCR and Western blot. The full-length recombinant human uPA cDNA (NM_002658.4;2398 bp) in the plasmid pReceiver-M15-uPA or control vector pReceiver-M15 (eYFP and neomycin as theselection markers) were purchased from GeneCopoeia, Inc.(MD). The A549 and H322 cells were transfected with pReceiver-M15-uPA or control vector by using the polyJet DNA transfection reagent (SignaGen, Laboratories, Gaithersburg, MD) according to themanufacturer's instructions and selected by neomycin (G418, 50–100 μg/ml) over two weeks. The regulatory efficacy of uPA was confirmed using Western blot analysis.

### Reverse transcription-polymerase chain reaction (RT-PCR)

Total mRNA from the lung adenocarcinoma cell lines was extracted using TRIzol reagent (Invitrogen) according to the manufacturer'sprotocol. 1 μg of mRNA was reverse transcribed to cDNA using Taqman reverse-transcription reagents (TAKARA, Japan). The primer sequences for the cancer invasion-related genes, including VEGFA, MMP2, TERT, PKLR, IGF1, RHOC, TGFB1, uPA, Id1, NFκB-p65, ERK1/2, IL1RL1, TWIST1, and ADAMTS1, are listed in Supplementary Table 1. The PCR procedure for amplification was 95°C for 30 s, followed by 32 cycles of 94°C for 30 s, 50°C (uPA, IGF1, IL1RL1,PKLR, MMP2, TERT) or 55°C (VEGFA, NFκB-p65, TGFβ-1, ERK1/2, Id1, TWIST, ADAMTS1, RHOC, KLF17) for 30 s, and 72°C for 30 s. β-actin was used as a loading control.

### Western blot analysis

Western blot was performed according to a previously described method [32] with antibodies for anti-human KLF17 (1:200, Santa Cruz, CA; 1:200, Sigma, CA), anti-uPA (1:200, Sigma, CA), anti-Phospho-p38/MAPK, anti-Phospho-pERK1/2, anti-Phospho-pSAPK/JNK, anti-Phospho-Akt, anti-Phospho-p44/42/MAPK, anti- Phospho-PI3-Kinasep85 and anti-Phospho-Src (Tyr527) (Cell Signaling Tech., Danvers, MA). Anti-GAPDH (Bioworld Tech, Inc., Nanjing, China) was used to detect the internal control.

### Invasion assay and rescue assay

The Matrigel-based invasion assay was performed as previously described [33]. Briefly, 2×10^4^ cells were plated in the upper Transwell chamber (Corning, NY, USA) in DMEM containing 1% FBS. The chamber basement membranewas coated with 60 μg Matrigel (Matrigel, Becton-DickinsonBiosciences, NJ). Then, the chamber was inserted into 24-well plates filled with 600 μL of DMEM medium containing 10% FBS. After incubation at 37°C for 24 h, the cells on the upper portion of the chamber membranes were removed, and the lower surfaces of the chamber membranes were fixed with 5% glutaraldehyde for 5 min and stained with 5% Giemsa. The number of invading cells from four randomly selected fields was counted under a microscope. For rescue assays, the A549 and H322 cells with stable expression of KLF17 protein were again transfected with pReceiver-M15-uPA containing uPA cDNA or a control vector; For effects of Src and p38/MAPK inhibition on invasion assay, the upper Transwell chamber (Corning, NY, USA) in DMEM containing 1% FBS and different concentration of SB 203580 (p38/MAPK inhibitor) or HY-13805 (PP2, Src inhibitor), and then the invasiveness of the cells was analyzed by Transwell assays in the same manner.

### Dual-luciferase reporter assay

The 3.1 kb promoter sequences of uPA was amplified from 293T cells and cloned into the pGL3-Basic (Promega) vector atthe *Kpn*I and *Xho*I sites. Additionally, different mutations of the uPA promoter sequences were ligated into the pGL3-Basic vector in the same manner, which were named as pGL3-uPA (−2950/+200 bp) and pGL3-uPA (−2000 /+200 bp). pcDNA3.1(+) mock vector was purchased from Addgene, and the pcDNA3.1(+)-KLF17 overexpression vector was cloned from Lentivirus vector pLV.0-KLF17. For the reporter assays, The pGL3-Basic and different pGL3-uPA mutant vectors were co-transfected into HEK-293FT cells in 24-well plates with pcDNA3.1(+)-KLF17 or negative control vector (pcDNA3.1(+)) and Renilla plasmid (20 ng) using Lipofectamine 2000 (Invitrogen). Firefly and Renilla luciferase activities were measured using a dual-luciferase assay (Promega, Madison, WI, USA) 24 h after transfection. The firefly luciferase activity was analyzed as the firefly/Renilla ratio.

### Tissue samples and immunohistochemistry

The 43 human lung samples were obtained from the First Affiliated Hospital of Jinan University with informed consent from the patients. The clinical characteristics of the patients were listed in Supplementary Table 2. Serial 4-μm-thick sections were used to analyze KLF17 and uPA expression by immunohistochemistry according to the protocols previously described [23, 32]. Staining intensity was graded according to the following scale: 0, negative; 1+, weak; 2+, moderate; and 3+, strong staining. Staining extent (percentage of stained cells) was scored into the following four categories: 1, 0% to 10%; 2, 11% to 50%; and 3, 51% to 100%. The immunostaining score was calculated by staining intensity×staining extent [51]. The optimal cut-off values of KLF17 or uPA were chosen based on statistical analysis using the log-rank test according to overall survival. In our study, staining index scores of ≥ 6 and ≥ 9 were regarded as optimal cut-off values for KLF17 and uPA expression levels, respectively.

### Statistical analysis

All experimental data were analyzed by using the SPSS statistical software (version 16.0; SPSS, Inc., Chicago, IL, USA). Student's *t-test* was used to analyze the results of the RT-PCR, Western blot and Transwell assay. All of the data are represented as the mean ± s.d. In addition, the comparison of survival times between the high- and low- KLF17 or uPA expression groups was performed by the Kaplan-Meier analysis and the log-rank test. The IHC staining score between two groups was compared by the Wilcoxon rank-sum test. ROC curve analysis was used to analyze the predictive value for lymph node metastasis of lung adenocarcinoma. *P* < 0.05 was considered to be statistically significant.

## SUPPLEMENTARY FIGURES AND TABLES


