# Supplementary material for: Krüppel-like factor 17 inhibits urokinase plasminogen activator gene expression to suppress cell invasion through the Src/p38/MAPK signaling pathway in human lung adenocarcinoma
Source: Oncotarget. 2017 Apr 10;8(24):38743–54. doi: 10.18632/oncotarget.17020 (PMC5503568; doi:10.18632/oncotarget.17020)
Supplement: Supplementary file 1 [file oncotarget-08-38743-s001.pdf]

Krüppel-like factor 17 inhibits urokinase plasminogen activator gene expression to suppress cell invasion through the Src/p38/MAPK signaling pathway in human lung adenocarcinoma

Supplementary Materials

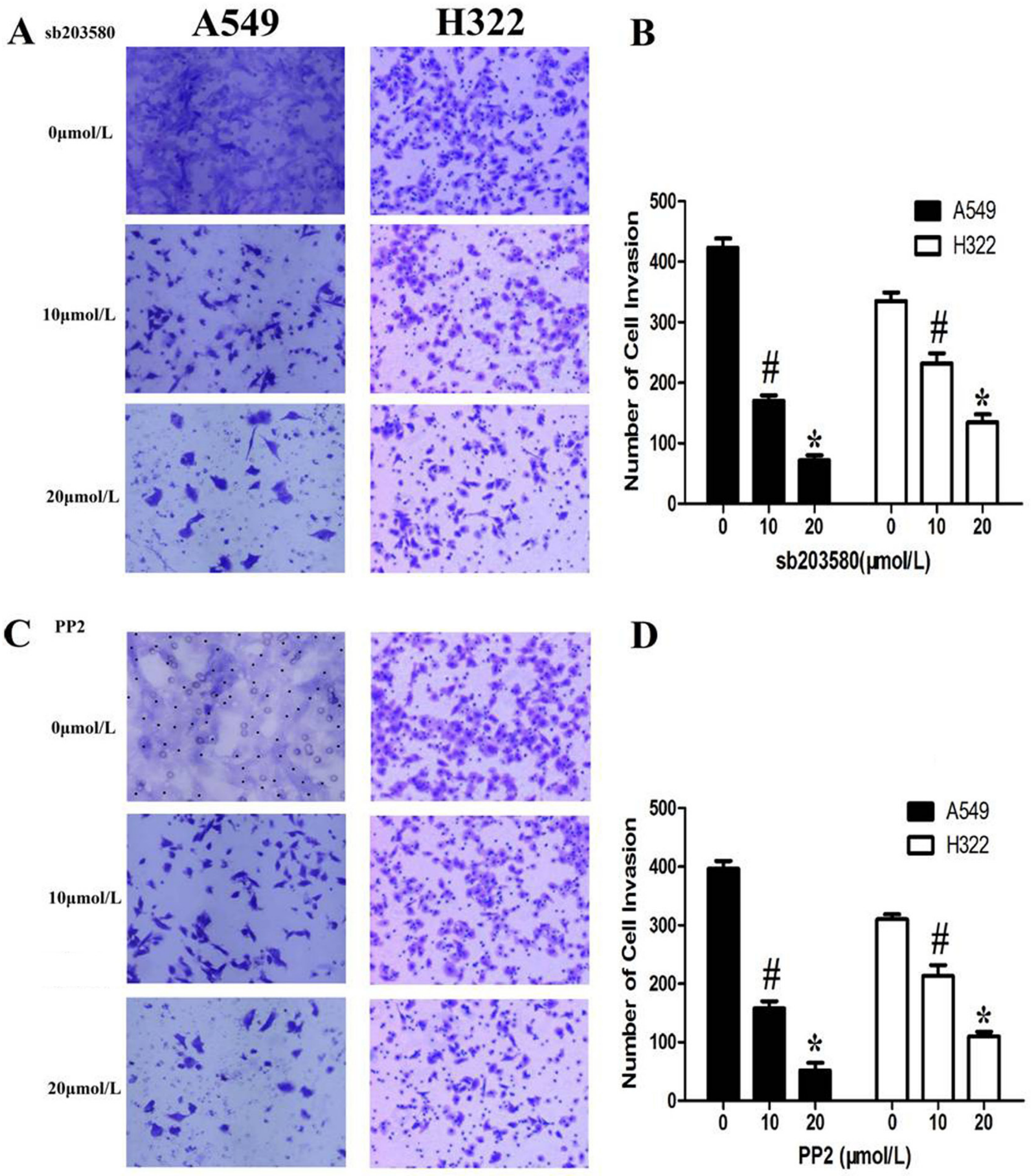

Supplementary Figure 1: Inhibition of p38/MAPK and Src pathway suppressed the invasion of A549 and H322 cells. (**A–C**) Representative areas of invaded cells by the different concentration of SB 203580 (p38/MAPK inhibitor) and HY-13805 (PP2, Src inhibitor) on A549 and H322 cells in the Transwell assay. (**D**) The graphs show the number of invaded A549 or H322 cells/field. Quantitative data represent means and SD of three independent experiments. $^{\#}P$ value < 0.01 compared with 0μmol/L group; $^{*}P$ value < 0.01 compared with 10 μmol/L group.

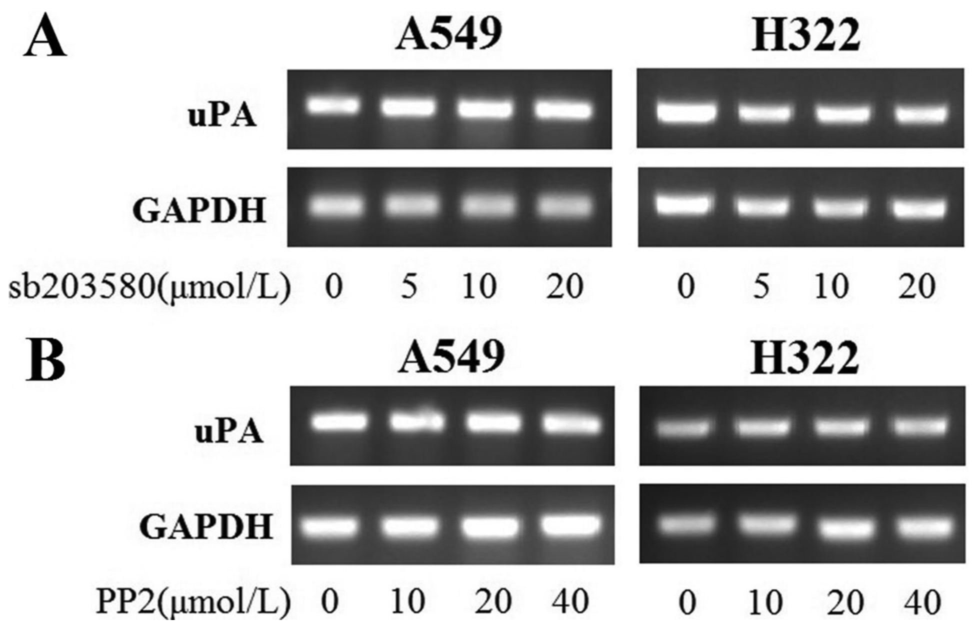

Supplementary Figure 2: IEffects of p38/MAPK and Src inhibitor on the mRNA expression of uPA in A549 and H322 cells. (**A, B**) RT-PCR showed that the mRNA expression of uPA were no difference among the different concentration of p38/MAPK and Src inhibitor groups.

Supplementary Table 1: The primers for target gene

Gene	primer	products
**VEGF**	Forward: ATCACGAAGTGGTGAAGTTC	265 bp
	Reverse: TGCTGTAGGAAGCTCATCTC	
**NFκB-P 65**	Forward: CCCCACGAGCTTGTAGGAAAG	91 bp
	Reverse: CCAGGTTCTGGAAACTGTGGAT	
**TGFβ1**	Forward: ACAAGTTCAAGCAGAGTACAC	161 bp
	Reverse: GCTGTATTTCTGGTACAGCTC	
**ERK1**	Forward: TCAACACCACCTGCGACCTT	102 bp
	Reverse: GCGTAGCCACATACTCCGTCA	
**ERK2**	Forward: GTTCCCAAATGCTGACTCCAA	126 bp
	Reverse: CTCGGGTCGTAATACTGCTCC	
**MMP2**	Forward: ATGGATCCTGGCTTTCCC	165 bp
	Reverse: GCTTCCAAACTTCACGCTC	
**ST2**	Forward: AGGCTTTTCTCTGTTTCCAGTAATCGG	659 bp
	Reverse:CAGTGACACAGAGGGAGTTCATAAAGTTAGA	
**IGF-1**	Forward: TCCTCGCATCTCTTCTACCTG	163 bp
	Reverse: TGCTGGAGCCATACCCTGT	
**uPA**	Forward: AGGACTACATCGTCTACCTG	199 bp
	Reverse: CAGATGGTCTGTATAGTCCG	
**Id1**	Forward: CAAGGTGAGCAAGGTGGAGATTC	450 bp
	Reverse: GCTTCAGCGACACAAGATGCG	
**TWIST1**	Forward: GAGACCTAGATGTCATTGTTTC	249 bp
	Reverse: GTTCAGACTTCTATCAGAATGC	
**PKLR**	Forward: TGATGAAATCCTGGAGGTGA	152 bp
	Reverse: GCATCTGTGTGGCACAGACA	
**ADAMTS1**	Forward: CAGAGCACTATGACACAGCA	180 bp
	Reverse: ACACGTGGCCTAATTCATGG	
**RHOC**	Forward: AGCCTGGAAAACATTCCTGAG	202 bp
	Reverse: CAAGGTAGCCAAAGGCACTG	
**TERT**	Forward: AGCCAGTCTCACCTTCAAC	187 bp
	Reverse: CAGCACACATGCGTGAAAC	
**KLF17**	Forward: GCTCTGGAGTGCACACCTCTT	74 bp
	Reverse: CAGCATCTCTGCGCTGTGA	
**β-actin**	Forward: TCCTTCCTGGGCATGGAGT	190 bp
	Reverse: TGATCTTCATTGTGCTGGGT	

Supplementary Table 2: The clinical characteristics of 43 patients with lung adenocarcinoma

Characteristics	*n* (%)	Characteristics	*n* (%)
Age (year)		IV	4(9.3)
≤ 65	25(58.1)	T stage	
> 65	18(41.9)	T1	18(41.9)
Gender		T2	20(46.5)
Male	30(69.8)	T3	2(4.7)
Female	13(30.2)	T4	3(7.0)
Smoking		N stage	
Yes	23(57.5)	N0	27(62.8)
No	20(52.5)	N1	7(16.3)
Tumor location		N2	8(18.6)
Left upper lobe	8(18.6)	N3	1(2.3)
Left lower lobe	5(11.6)	M stage	
Right upper lobe	15(34.9)	MO	39(90.7)
Right middle lobe	4(9.3)	M1	4(9.3)
Right lower lobe	11(25.6)	KLF17 expression	
Differentiation		High expression	17(39.5)
Poor or lower	18(41.9)	Low expression	26(60.5)
moderate	20(46.5)	uPA expression	
well	5(11.6)	High expression	23(53.5)
Pathological stage		Low expression	20(46.5)
IA/B	27(62.8)	Vital status	
IIA/B	5(11.6)	Death	12(27.9)
IIIA/B	7(16.3)	Alive	31(72.1)
